# Computational modeling of fear and stress responses: validation using consolidated fear and stress protocols

**DOI:** 10.3389/fnsys.2024.1454336

**Published:** 2024-12-24

**Authors:** Brunna Carolinne Rocha Silva Furriel, Geovanne Pereira Furriel, Mauro Cunha Xavier Pinto, Rodrigo Pinto Lemos

**Affiliations:** ^1^Instituto Federal de Goiás, Goiânia, Brazil; ^2^Universidade Federal de Goias, School of Electrical, Mechanical and Computer Engineering, Goiânia, Brazil; ^3^Imaging Research Center, Hospital Israelita Albert Einstein, São Paulo, Brazil; ^4^Instituto Federal Goiano, Trindade, Brazil; ^5^Universidade Federal de Goias, Institute of Biological Sciences, Goiânia, Brazil

**Keywords:** computational modeling, neural architecture, contextual fear conditioning, fear extinction, stress models, stress-enhanced fear learning (SEFL), Immediate Extinction Deficit (IED), biologically plausible models

## Abstract

Dysfunction in fear and stress responses is intrinsically linked to various neurological diseases, including anxiety disorders, depression, and Post-Traumatic Stress Disorder. Previous studies using in vivo models with Immediate-Extinction Deficit (IED) and Stress Enhanced Fear Learning (SEFL) protocols have provided valuable insights into these mechanisms and aided the development of new therapeutic approaches. However, assessing these dysfunctions in animal subjects using IED and SEFL protocols can cause significant pain and suffering. To advance the understanding of fear and stress, this study presents a biologically and behaviorally plausible computational architecture that integrates several subregions of key brain structures, such as the amygdala, hippocampus, and medial prefrontal cortex. Additionally, the model incorporates stress hormone curves and employs spiking neural networks with conductance-based integrate-and-fire neurons. The proposed approach was validated using the well-established Contextual Fear Conditioning paradigm and subsequently tested with IED and SEFL protocols. The results confirmed that higher intensity aversive stimuli result in more robust and persistent fear memories, making extinction more challenging. They also underscore the importance of the timing of extinction and the significant influence of stress. To our knowledge, this is the first instance of computational modeling being applied to IED and SEFL protocols. This study validates our computational model's complexity and biological realism in analyzing responses to fear and stress through fear conditioning, IED, and SEFL protocols. Rather than providing new biological insights, the primary contribution of this work lies in its methodological innovation, demonstrating that complex, biologically plausible neural architectures can effectively replicate established findings in fear and stress research. By simulating protocols typically conducted *in vivo*-often involving significant pain and suffering-in an insilico environment, our model offers a promising tool for studying fear-related mechanisms. These findings support the potential of computational models to reduce the reliance on animal testing while setting the stage for new therapeutic approaches.

## 1 Introduction

Fear and stress drive adaptive behavior in response to environmental challenges. The activation of fear and stress responses triggers a cascade of autonomic and endocrine changes, significantly impacting learning and memory processes, as shown in seminal neurology and psychology research (Squire, 1987, 2009; McGaugh, 2013). The effects of these changes depend on their timing relative to the learning process (Drexler et al., 2019).

Pavlovian fear conditioning has become an essential tool for investigating cognitive paradigms in both human and animal research. This methodology has significantly enhanced our understanding of the physiological basis of fear and has applications in preclinical models of neuropathologies and clinical research (Chang et al., 2009). Through pre-training, post-training, and pre-test manipulations, Pavlovian fear conditioning provides insights into the complexities of memory acquisition, consolidation, and retrieval (LeDoux, 2000; Maren, 2011).

Fear conditioning involves associating a neutral stimulus or context with an unconditioned stimulus (*US*), resulting in the neutral stimulus acquiring aversive properties and becoming a conditioned stimulus (*CS*). This process elicits conditioned responses (*CR*), a well-documented phenomenon (Ehrlich et al., 2009). Additionally, extinction is introduced as a context-dependent learning form, describing the reduction of conditioned responses when the *CS* is presented without the *US*, leading to the suppression, but not erasure, of the memory (Turnock and Becker, 2008; Chang and Liang, 2017).

The efficacy of Pavlovian fear conditioning depends on the strength of the conditioned-unconditioned stimulus pairing and can be reversed during extinction processes. These limitations pose challenges in elucidating the mechanisms underlying stress and anxiety, affecting the development of effective behavioral therapies for related disorders (Maren et al., 2013; LeDoux, 2014; Maren and Holmes, 2016; Bennett et al., 2019). Consequently, stress models such as Immediate Extinction Deficit (IED) and Stress-Enhanced Fear Learning (SEFL) have been developed to understand stress's influence on fear memory.

The Stress-Enhanced Fear Learning (SEFL) model aims to enhance our understanding of disorders like Post-Traumatic Stress Disorder (PTSD). It focuses on how traumatic experiences affect learning responses, such as freezing in rats exposed to shocks in various contexts (Rau et al., 2005). This model emphasizes sensitization and generalization in fear learning following trauma (Long and Fanselow, 2012).

In contrast, the Immediate Extinction Deficit (IED) model investigates how stress affects the ability to “unlearn” fear. It shows that animals exposed to extinction training shortly after conditioning exhibit different recovery patterns depending on the training's timing (Kim et al., 2010; Maren, 2014). This model underlines the impact of timing on extinction learning effectiveness.

The need to develop more effective treatments for neurological diseases related to fear and stress drives the search for a deeper understanding of these mechanisms. Conventional in vivo experiments provide valuable information but face significant limitations, including ethical concerns and the risk of causing trauma or exacerbating preexisting conditions. This highlights the need for new approaches and technologies. In this context, stress models and computational tools are valuable resources, allowing detailed analysis of the neural mechanisms associated with fear and stress. Computational modeling, in particular, enables the simulation and understanding of the complex dynamics between fear, stress, and related disorders (Yamamori and Robinson, 2023).

Despite biological and cognitive differences between rodents and humans, using a rodent neural architecture is justified by the extensive research in the literature, facilitating comparisons with preexisting models and providing a robust foundation for validation and further insights (Morén, 2001; Moustafa et al., 2009; John et al., 2013; Pendyam et al., 2013; Feng et al., 2016; Li, 2017; Mattera et al., 2020; Khalid et al., 2020; Turnock and Becker, 2008; Chang and Liang, 2017; McGaugh, 2015; Okon-Singer et al., 2015; Li, 2017; Kahana, 2020).

Thus, this study aims to develop a biologically and behaviorally plausible computational framework based on a rodent brain to analyze responses to fear and stress through fear conditioning, IED, and SEFL approaches. The primary goal is to construct a computational model representing the neural properties of critical brain structures involved in fear processing, including subregions of the amygdala, hippocampus, prefrontal cortex, nucleus reuniens, and dynamic stress hormone responses. By incorporating greater structural complexity and specific synaptic parameters, this approach seeks to validate the model's robustness through its ability to replicate established findings in the literature. We conducted experiments to assess the model's capability to reflect physiological and behavioral characteristics, ultimately establishing a credible foundation for future *in silico* studies that can potentially reduce animal testing needs.

## 2 Methods

### 2.1 Model overview

The proposed architecture integrates several subregions of crucial brain structures, such as the amygdala, hippocampus, medial prefrontal cortex, and nucleus reuniens. The model was developed based on rats' neurobiological parameters, ensuring that all data regarding neural architecture, synaptic weights, signal propagation, and other variables are consistent with studies in this species. This choice aligned the model with widely used fear conditioning protocols, such as Contextual Fear Conditioning, SEFL, and IED, which traditionally employ rats.

Furthermore, it proposes an innovative computational model incorporating stress hormone curves and utilizing firing neural networks with conductance-based integrating and firing neurons. We employed the well-established paradigm of Contextual Fear Conditioning for model initial validation. Subsequently, we used the IED and SEFL protocols to evaluate the model's applicability in studying disorders related to fear and stress.

**Figure 1:**
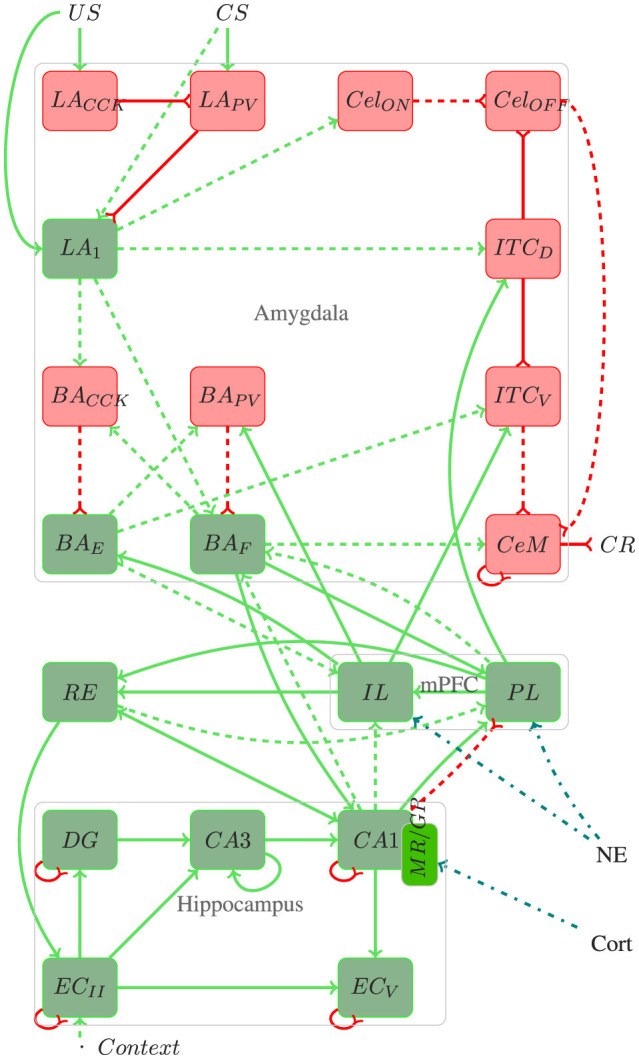
Architecture of the proposed model. Red rectangles and lines represent inhibitory connections, green rectangles and lines represent excitatory connections, blue lines represent stress responses, and dotted lines represent plastic connections. In addition to the elucidated information within the text, pertinent details regarding the number of neurons, connections, and referenced works for each region utilized in the proposed model are provided throughout the text.

A graphical representation of the model's architecture is presented in Figure 1. The detailed parameters of the model, including the configuration of the integrate-and-fire (IF) neurons, the synaptic weights, and the input connections for each implemented neuronal cluster, are documented below.

The amygdala plays a fundamental role in forming and extinction fear memory, acting as a key processing center for *CS*−*US* stimuli and integrating sensory information to influence executive, motor, and memory functions (Akirav and Maroun, 2007; Carrere and Alexandre, 2015). Its sensory input region, the lateral amygdala (*LA*), receives projections from various cortices, including auditory, visual, gustatory, olfactory, and somatosensory, and is crucial for responding to both conditioned (*CS*) and unconditioned stimuli (*US*) (Connor and Gould, 2016).

In the *LA*, excitatory neurons (*LA*) coexist with inhibitory neurons containing somatostatin (*LA*_*SOM*_), parvalbumin (*LA*_*PV*_), and cholecystokinin (*LA*_*CCK*_). The *LA* neurons, receiving both *CS* and *US* inputs, play a pivotal role in encoding fear memories, while *LA*_*PV*_ and *LA*_*CCK*_ modulate these responses through their inhibitory projections (Duvarci and Pare, 2014; Kim et al., 2013; Bennett et al., 2019).

The medial central amygdala (*CeM*) orchestrates the behavioral, autonomic, and endocrine responses associated with fear, receiving inputs from the *LA* through various pathways. These include the basal amygdala (*BA*) pathway, critical for transmitting *LA* activity to *CeM* (Pape and Pare, 2010; Asede et al., 2015; Pare and Duvarci, 2012), and the intercalated inhibitory cells, which modulate fear responses during both acquisition and extinction phases (Duvarci and Pare, 2014; Oliva et al., 2018). The lateral nucleus of the amygdala (*LA*) neurons excite neurons within the basolateral nucleus (*BA*), which are divided into two distinct sub-populations: one associated with fear acquisition (*BA*_*F*_) and the other with extinction (*BA*_*E*_) (Herry et al., 2008). Also within the basolateral region are inhibitory neurons containing parvalbumin (*BA*_*PV*_) and cholecystokinin (*BA*_*CCK*_). Additionally, the lateral subdivision of the central amygdala (*CeL*) regulates the output of *CeM* cells, influenced by both *US* and *LA* projections (Ciocchi et al., 2010; Mattera et al., 2020; Haubensak et al., 2010; Duvarci and Pare, 2014).

Another critical region of the fear circuit is the medial prefrontal cortex (*mPFC*). Studies indicate that fear memory extinction requires plasticity in the mPFC and the amygdala (Akirav and Maroun, 2007). The *mPFC* can modulate the expression of previously learned fear bidirectionally, i.e., it performs coordinated action by integrating several mnemonic inputs and up-down regulation of specific brain circuits (Gilmartin et al., 2014).

The medial prefrontal cortex (*mPFC*) also contributes significantly to the fear circuit, modulating the expression of learned fear through its connections with the amygdala (Gilmartin et al., 2014; Akirav and Maroun, 2007). The infralimbic (*IL*) and prelimbic (*PL*) cortices are integral components of the *mPFC*, crucial for both the formation and extinction of fear memories. While the *PL* primarily contributes to fear acquisition, the *IL* is primarily involved in fear extinction (Marek et al., 2018b). Additionally, they exert top-down regulation on the fear response (Sierra-Mercado et al., 2011; Bennett and Lagopoulos, 2018; Marcus et al., 2020).

Furthermore, the hippocampus and the entorhinal cortex (*EC*) are integral for contextual fear memory processing, communicating through both the trisynaptic (TSP) and monosynaptic (MSP) pathways. These regions send emotion-related information to the amygdala and *mPFC*, influencing the encoding and recall of emotional memories (Schapiro et al., 2017; O'Reilly and Norman, 2002; Ketz et al., 2013; Tse et al., 2007; Maren et al., 2013; OReilly et al., 2014).

The nucleus reuniens (*RE*) connects cortical structures and the hippocampus, significantly influencing contextual fear learning and memories (Bokor et al., 2002; Vertes, 2006). Inactivation of the RE affects acquiring and retrieving these memories, while projections from the medial prefrontal cortex (*mPFC*) to the RE are essential for inhibiting fear after extinction (Ramanathan et al., 2018).

### 2.2 Spiking neural networks

The cells of the proposed model use mainly Integrated and Fire (IF) type neurons based on conductance to express the firing dynamics coming from each network layer (Destexhe, 1997). The IF artificial neuron is a model capable of expressing the dynamics of the Spiking Neural Network (SNN), which describes mathematically the properties of biological neurons that generate electrical potential through the cell membrane caused by the change in the conductance of the receptor channel in the presynaptic region (Destexhe, 1997; Gerstner et al., 2014; Raudies and Hasselmo, 2014; Rezaei et al., 2020).

The IF model analyzes neuron action potential propagation through a time-dependent current. When the potential reaches a certain established threshold, it triggers spikes, instantly raising the potential before it returns to its resting value (Abbott, 1999). According to the model, the membrane potential is given by:


(1) $$\begin{array}{l} C\frac{dV_{i}}{dt}=-g_{leak}\left[V_{i}\left(t\right)-E\right]+I_{syn}\left(t\right)+\eta \end{array}$$

The input current *I*_*syn*_ drives the membrane, modeled using capacitance *C* with potential *V*_*i*_, through the leakage conductance channel *g*_*leak*_, where *E* represents the synaptic conductance reversal equilibrium potential. The index *i* represents the *i*_*th*_ modeled region. The term η represents small fluctuations in the membrane potential and is a random variable η* ϵN*(μ, σ), extracted from the Gaussian distribution *N* with mean value μ and standard deviation σ.

The synaptic current is modeled as the ohmic conductance *g*_*syn*_ multiplied by the driving force, which is the difference between the membrane potential *V*_*i*_ and the reversal equilibrium potential of the synaptic conductance *E*_*syn*_ (Destexhe, 1997).


(2) $$\begin{array}{l} I_{syn}\left(t\right)=+g_{syn}\left(t\right)\left[E_{syn}-V_{i}\left(t\right)\right] \end{array}$$

Including the excitatory electrical conductivities, *g*_*E*_, and inhibitory electrical conductivities, *g*_*I*_, and considering the membrane potential about the τ refractory period, the membrane potential is given by:


(3) $$\begin{array}{l} \tau \frac{dV_{i}}{dt}=\left[E-V_{I}\left(t\right)\right]+\frac{g_{E}}{g_{leak}}\left[E_{E}-V_{I}\left(t\right)\right]+\frac{g_{I}}{g_{leak}}\left[E_{I}-V_{I}\left(t\right)\right]+\eta \end{array}$$

when $\tau =\frac{C}{g_{leak}}$. Furthermore, it has different values for each type of neuron. *E*_*E*_ represents the excitatory reversal equilibrium potential, and *E*_*I*_ is the inhibitory potential.

When the membrane potential reaches the membrane potential threshold, the neuron fires rise to the peak potential, *V*_*th*_, and then returns to the resting potential.


(4) $$\begin{array}{l} \begin{array}{cc} V_{i}\to V_{reset} & if\left(V_{i}\left(t\right)>V_{th}\right) \end{array} \end{array}$$

To update the synaptic weights of IF, we used Spike Time Dependent Plasticity (STDP), an adapted form of Hebbian learning, and frequently implemented in SNN. In the biological context, synaptic plasticity is divided into Long Term Potentiation (LTP) and Long Term Depression (LTD), where LTP represents the changes when a synaptic increase occurs and LTD, a decrease in synaptic gain. STDP suggests that synaptic efficacy increases when presynaptic peaks occur milliseconds before postsynaptic peaks. Likewise, the efficacy decreases when postsynaptic peaks occur before presynaptic peaks (Gupta and Long, 2009).

The model uses a learning mechanism based on physiological data to analyze forwarding and backward temporal order repetition. The weight adaptation is given by:


(5) $$\tau_{\omega }\frac{d\omega }{dt}=\{\begin{array}{ll} (\omega_{max}-\omega )A_{+}\cdot exp\left(\frac{-\text{Δ}t}{\tau_{+}}\right) & if\;\text{Δ}t\leq 0\\ (\omega_{min}-\omega )A_{-}\cdot exp\left(\frac{+\text{Δ}t}{\tau_{-}}\right) & if\;\text{Δ}t>0 \end{array}$$

Δ*t* = (*t*_*pre*_−*t*_*pos*_) and the relative time between the presynaptic and postsynaptic peak, wherein positive Δ*t* represents LTP and negative, LTD. ω_*min*_ and ω_*max*_ refer to lower and upper bounds of the dynamic range of weights and the time constants τ_ω_, τ_+_ and τ_−_ are weight adaptation and LTP and LTD learning curve, respectively. Finally, *A*_+_ and *A*_−_ represent amplitude for depression and synaptic potentiation. In a computational model, *A*_+_ is three times greater than *A*_−_.

The existing physiological evidence justifies STDP, showing that LTD is necessary for context-based learning (Raudies and Hasselmo, 2014). This method effectively defines time intervals and peak frequencies within the current Theta phase oscillation ranges, observed in coding and evocation processes in the hippocampus and the amygdala.

### 2.3 Sparsity

In our model, sparsity refers to the number of neurons remaining active within a given layer, controlled by the k-Winner Take All (k-WTA) function. This function limits the number of active neurons by applying a global inhibitory value across the entire layer, allowing only the top *k* neurons with the highest excitation levels to remain active while suppressing the others. As activity flows through the layers, excitatory and inhibitory weights (ω_*exc*_ and ω_*inib*_) interact to regulate this process, with the k-WTA function enhancing inhibition by activating the most substantial peaks and preventing other neurons from firing (Smith, 2020).

This sparsity mechanism is represented by generating a pulse of current *I* based on the k-WTA function:


(6) $$\begin{array}{l} I_{j}=argmax_{j}\overset{n_{k}}{\underset{i=1}{\sum }}\left(V_{i}-E\right)\omega^{exc}-\overset{n_{k}}{\underset{i=1,i\neq j}{\sum }}\left(V_{i}-E\right)\omega^{inib} \end{array}$$

This approach results in a distributed activation in layers such as those in the hippocampus, where a subset of neurons remains active rather than just a single dominant neuron. The degree of sparsity in these layers can vary based on the model's stress level, with higher stress potentially leading to a more significant proportion of active neurons. Conversely, only the most active neuron is triggered in layers focusing on a single dominant signal. This approach mirrors natural neural systems, where only a select group of neurons respond robustly to specific inputs, ensuring that only the most relevant signals propagate through the network while balancing excitatory and inhibitory activity.

### 2.4 Neuromodulation

In response to a stressful event, the body activates the sympathetic nervous system and the hypothalamic-pituitary-adrenal (HPA) axis, triggering a cascade of biochemical reactions to handle the situation (Drexler et al., 2019; Grzelka et al., 2017). This response prepares the body for immediate action and medium-term adjustment, which is necessary to cope with stress's physiological and psychological impact.

During the stress response, neurotransmitters and hormones play essential roles in modulating neural activity. They enhance the organism's readiness and reactivity and regulate processes related to memory and learning, which are critical in forming fear responses (Krugers et al., 2012; Wolf, 2017). The interaction between these neurotransmitters and hormones allows the brain to process the stressful event and adjust neural activity according to the intensity and duration of the stimulus.

The main agents involved in the stress response include norepinephrine and corticosteroids. Norepinephrine primarily acts as a neurotransmitter in the brain, quickly affecting neural circuits to intensify alertness and immediate readiness. In contrast, corticosteroids function as longer-acting hormones that gradually modulate neural activity (Joëls et al., 2006; Taborsky et al., 2020). This temporal difference allows norepinephrine and corticosteroids to work together to adapt the organism to stress: norepinephrine impacts the initial response of arousal and vigilance, while corticosteroids contribute to stress regulation and long-term memory consolidation.

**Figure 2:**
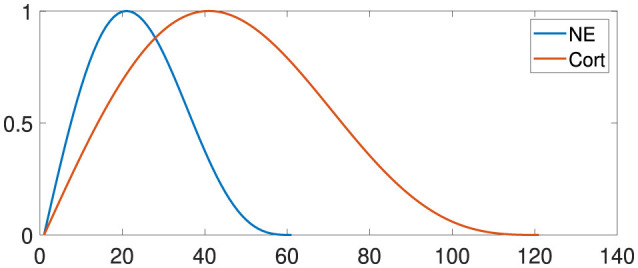
Neuroendocrine responses to stress. Noradrenaline (NE) levels increase until approximately twenty minutes and decrease until sixty minutes. Corticosteroid levels also increase exponentially and return to baseline after one to 2 h. The x-axis specifies the time in minutes.

To model neuromodulation in this study, we used simplified response equations and curves for norepinephrine and corticosteroids following a stressful event, based on the foundational work of Krugers et al. (2012) and Wolf (2017). Figure 2 illustrates these levels, with adjustments made to capture each hormone's distinct temporal patterns and intensity levels: norepinephrine rises quickly to support immediate alertness, while corticosteroids increase gradually to sustain a prolonged regulatory effect. Although simplified, these curves capture the primary trends in hormonal response timing and progression, providing a practical yet realistic approach to modeling neuromodulatory effects.

The action potential of layers with neurotransmitter interference is multiplied by a value representing the tonic level β.


(7) $$\begin{array}{l} \beta_{NE}=0.3853\cdot sen\left(\frac{2\cdot \pi \left(t\right)}{60}\right)+0.7706\cdot abs\left(sen\left(\frac{2\cdot \pi \left(t\right)}{60}\right)\right) \end{array}$$

(8) $$\begin{array}{l} \beta_{CORT}=0.3853\cdot sen\left(\frac{2\cdot \pi \left(t\right)}{120}\right)+0.7706\cdot abs\left(sen\left(\frac{2\cdot \pi \left(t\right)}{120}\right)\right) \end{array}$$

To illustrate the impact of NE-influenced neuromodulation on the *PL* and *IL* regions, all inputs are scaled by the factor β. Thus, the input *I*_*j*_ is determined as follows:


(9) $$\begin{array}{l} I_{inf}=\overset{n-inf}{\underset{inf=1}{\sum }}\left(V_{i}-E\right)\omega_{inf} \end{array}$$

(10) $$\begin{array}{l} I_{j}=arg\;max_{j}\{\left(1+\beta \right)\cdot I_{inf}+\sum_{i=1}^{n_{k}}\left(V_{i}-E\right)\omega^{exc}\\ \;-\sum_{i=1,i\neq j}^{n_{k}}\left(V_{i}-E\right)\omega^{inib}\} \end{array}$$

where *I*_*inf*_ is the hormonal influence.

To represent the influence of corticosteroids in the *CA*1 region and the consequent increase in the firing of neurons, the percentage of the sparseness of the *CA*1 region may vary from 10%, standard value, to 50%, saturation value at 40 min after the stressful situation.

### 2.5 Experimental design

Each experiment is divided into several groups, each performing a specific set of tasks. The timing of behavioral episodes is divided into several phases. In each phase, different initial stimuli trigger unique behaviors or reactions. Furthermore, several cycles of repetition of these behaviors or reactions may occur within each phase. The Table 1 details the correlation between the expressions, the number of cycles and their input stimuli.

**Table 1:** Relationship between expressions and input stimuli.

**Expression**	**Input Stimuli**		
	**Context**	**Sound**	**Shock**
Home	Home	No	No
A	A	No	No
B	B	No	No
C	C	No	No
AX–	A	Yes	No
BX–	B	Yes	No
CX–	C	Yes	No
AX+	A	Yes	Yes
BX+	B	Yes	Yes
CX+	C	Yes	Yes

The contexts are *A*, *B*, *C*, and *Home*. *X* represents the presence of the sound stimulus, and the symbols + and − represent the presence or absence of *US* (shock), respectively. For example, *AX*+ represents context *A*, with sound and shock.

Before initiating any experiment, we establish the computational model's initial configuration through a simulation in the “Home” environment. This process is essential to ensure the neural network's dynamic equilibrium and to prevent any bias in the experimental responses. During this initial phase, we calibrate the synaptic weights, which are adopted as baseline parameters for all subsequent experiments. The simulation in the “Home” environment continues until the output of the central nucleus of the amygdala (*CeM*) stabilizes below 10% for ten consecutive cycles. This procedure ensures that the network has achieved a state of consistency and robustness, allowing experiments to be conducted with the assurance that the baseline freezing behavior has been adequately controlled and stabilized.

We initialize neurons with a resting potential of −70 × 10^−3^*V*, and adjust the membrane potential at each cycle as previously described. After completing the final phase of each experiment, we analyze neuronal activity in the *CeM* to interpret the network's prediction.

We initiate stress cycles after fifteen cycles of fear acquisition. We selected this threshold of 15 cycles based on baseline factors to activate the norepinephrine and corticosteroid response curves, establishing a reasonable timeframe for stress hormones to begin interacting with the fear acquisition process. Given the various approaches for modeling fear acquisition and transitioning to stress, we chose the 15-cycle mark to introduce stress cycles in a way that reflects a plausible temporal dynamic between fear and stress mechanisms. We adjust stress levels according to Equations 7, 8.

The developed computational model incorporates shock intensity as a configurable variable, enabling the simulation of different levels of unconditioned stimulus (*US*) intensity by practices observed in behavioral studies. This parameterization allows for replicating variations in behavioral responses found in *in vivo* experiments.

Additionally, the model maintains a fixed time interval between shocks to ensure consistency in timing during each acquisition phase. The unconditioned stimulus (US) is applied in 2, 000 of the total 8, 000 iterations per phase, ensuring a uniform application of the stimuli. This stringent control of the interval between shocks reflects standard practices in *in vivo* experiments. Standardizing time between stimuli is essential to minimize variations that may interfere with behavioral responses.

To represent the propagation of the shock effect, the initial current in the sensory layers was set to 1.00*nA*, ensuring the proper transmission of the stimulus. Additionally, the synaptic current is reduced at a rate of 0.01*nA* per subsequent layer, simulating the decay of shock intensity as the stimulus progresses through the neural circuit, similar to what occurs in biological systems.

Parameters for configuring the IF neuron include the membrane capacitance (*C*) set to 5.5*pF*, and the membrane conductance (*G*_*l*_) set to 10*nS*, ensuring that the time constant (τ) is maintained at 0.5*ms*. The peak potential (*V*_*pico*_) is set to 0*mV*. The time interval and step for simulation of the computational model were defined to keep the network frequency close to 8 Hz, representing Theta oscillation. Thus, the time varies close to 125 ms. Each analysis is performed with an interval between 0 and 4, 000 ms and τ = 0.5 ms, resulting in 8, 000 cycles.

Synaptic weights must be modified until the network is appropriately converging to perform all training and testing. They are randomly initialized within the range of values determined by normal distribution, with standard deviation (σ) being 0.3 and mean (μ). Table 2 presents neurons number, connection, mean of the normal distribution, and the design references in each region used on the proposed model.

**Table 2:** Neurons number, connection, mean of the normal distribution, and the design references in each region used on the proposed model.

**Region**	**Neuron number**	**Connection**	**μ**	**Design references**
*EC*_*II*_	50	*Context*−*EC*_*II*_	1.2	O'Reilly and Rudy, 2001; Ketz et al., 2013; Maren et al., 2013; OReilly et al., 2014; Schapiro et al., 2017
				*RE*−*EC*_*II*_	3.0	Hoover and Vertes, 2007; Varela et al., 2014; Ramanathan et al., 2018; Dolleman-van der Weel et al., 2019; Bouton et al., 2021
*DG*	500	*EC*_*II*_−*DG*	1.2	O'Reilly and Rudy, 2001; Ketz et al., 2013; Maren et al., 2013; OReilly et al., 2014; Schapiro et al., 2017
*CA*_3_	160	*EC*_*II*_−*CA*3	1.2	O'Reilly and Rudy, 2001; Ketz et al., 2013; Maren et al., 2013; OReilly et al., 2014; Schapiro et al., 2017
				*DG*−*CA*3	1.2	O'Reilly and Rudy, 2001; Ketz et al., 2013; Maren et al., 2013; OReilly et al., 2014; Schapiro et al., 2017
*CA*_1_	40	*CA*3−*CA*1	1.2	O'Reilly and Rudy, 2001; Ketz et al., 2013; Maren et al., 2013; OReilly et al., 2014; Schapiro et al., 2017
				*BA*_*F*_−*CA*1	1.0	O'Reilly and Rudy, 2001; Ketz et al., 2013; Maren et al., 2013; OReilly et al., 2014; Schapiro et al., 2017
				*RE*−*CA*1	3.0	Hoover and Vertes, 2007; Varela et al., 2014; Ramanathan et al., 2018; Dolleman-van der Weel et al., 2019; Bouton et al., 2021
		*EC*_*V*_	20	*CA*_1_−*EC*_*V*_	1.2	O'Reilly and Rudy, 2001; Ketz et al., 2013; Maren et al., 2013; OReilly et al., 2014; Schapiro et al., 2017
				*EC*_*II*_−*EC*_*V*_	1.2	O'Reilly and Rudy, 2001; Ketz et al., 2013; Maren et al., 2013; OReilly et al., 2014; Schapiro et al., 2017
*IL*	20	*BA*_*E*_−*IL*	3.3	Courtin et al., 2014; Mattera et al., 2020; Cummings and Clem, 2020
				*CA*_1_−*IL*	1.2	Tierney et al., 2004; Wang et al., 2018; Marek et al., 2018a
				*PL*−*IL*	1.2	Marek et al., 2018b; Mattera et al., 2020
		*US*−*IL*	1.2	Mattera et al., 2020
*PL*	20	*BA*_*F*_−*PL*	2.2	Gabbott et al., 2006; Bennett et al., 2017; Oliva et al., 2018
				*RE*−*PL*	2.0	Hoover and Vertes, 2007; Varela et al., 2014; Ramanathan et al., 2018; Dolleman-van der Weel et al., 2019; Bouton et al., 2021
				*CA*_1_−*PL*	0.6	Tierney et al., 2004; Sotres-Bayon et al., 2012; Wang et al., 2018
*RE*	20	*IL*−*RE*	3.0	Hoover and Vertes, 2007; Varela et al., 2014; Ramanathan et al., 2018; Dolleman-van der Weel et al., 2019; Bouton et al., 2021
				*PL*−*RE*	3.0	Hoover and Vertes, 2007; Varela et al., 2014; Ramanathan et al., 2018; Dolleman-van der Weel et al., 2019; Bouton et al., 2021
				*CA*1−*RE*	3.0	Hoover and Vertes, 2007; Varela et al., 2014; Ramanathan et al., 2018; Dolleman-van der Weel et al., 2019; Bouton et al., 2021
*LA*	50	*US*−*LA*	1.52	Romanski et al., 1993; Blair et al., 2001; Mattera et al., 2020
				*CS*−*LA*	0.35	Romanski et al., 1993; Blair et al., 2001; Mattera et al., 2020
				*LA*_*PV*_−*LA*	0.35	Wolff et al., 2014; Krabbe et al., 2018; Mattera et al., 2020
*LA*_*PV*_	10	*CS*−*LA*_*PV*_	0.5	Wolff et al., 2014; Krabbe et al., 2018; Mattera et al., 2020
				*LA*_*CCK*_−*LA*_*PV*_	1.8	Wolff et al., 2014; Krabbe et al., 2018; Rhomberg et al., 2018; Mattera et al., 2020
*LA*_*CCK*_	10	*US*−*LA*_*CCK*_	1.8	Wolff et al., 2014; Krabbe et al., 2018; Rhomberg et al., 2018; Mattera et al., 2020
*BA*_*CCK*_	10	*BA*_*F*_−*BA*_*CCK*_	1.9	Stefanacci et al., 1992; Pitkänen et al., 1995; Savander et al., 1997; Mattera et al., 2020
				*LA*−*BA*_*CCK*_	1.9	Stefanacci et al., 1992; Pitkänen et al., 1995; Savander et al., 1997; Mattera et al., 2020
*BA*_*E*_	50	*BA*_*CCK*_−*BA*_*E*_	0.7	Duvarci and Pare, 2014; Vogel et al., 2016; Mattera et al., 2020
				*IL*−*BA*_*E*_	3.3	Vertes, 2004; Cho et al., 2013; Courtin et al., 2014; Mattera et al., 2020
*BA*_*F*_	80	*LA*−*BA*_*F*_	5.0	Stefanacci et al., 1992; Pitkänen et al., 1995; Savander et al., 1997; Mattera et al., 2020
				*ITC*_*V*_−*BA*_*F*_	8.0	Asede et al., 2015
				*PL*−*BA*_*F*_	3.6	Vertes, 2004; Cho et al., 2013; Courtin et al., 2014; Mattera et al., 2020
				*BA*_*PV*_−*BA*_*F*_	2.0	O'Reilly and Rudy, 2001; Ketz et al., 2013; Maren et al., 2013; OReilly et al., 2014; Schapiro et al., 2017; Mattera et al., 2020
*BA*_*PV*_	10	*BA*_*E*_−*BA*_*PV*_	2.0	Bennett et al., 2017; Mattera et al., 2020
*ITC*_*D*_	20	*LA*−*ITC*_*D*_	1.0	Oliva et al., 2018		*ITC*_*D*_−*ITC*_*V*_	1.0	Duvarci and Pare, 2014; Oliva et al., 2018
		*BA*_*E*_−*ITC*_*V*_	1.0	Amano et al., 2010; Mattera et al., 2020
*CeL*_*ON*_	10	*LA*−*CeL*_*ON*_	0.8	Pape and Pare, 2010; Li et al., 2013; Mattera et al., 2020
*CeL*_*OFF*_	10	*CeL*_*ON*_−*CeL*_*OFF*_	0.2	Ciocchi et al., 2010; Duvarci and Pare, 2014; Oliva et al., 2018
				*ITC*_*D*_−*CeL*_*OFF*_	1.0	Bennett et al., 2019
*CeM*	2	*BA*_*F*_−*CeM*	1.9	Asede et al., 2015
				*ITC*_*V*_−*CeM*	1.0	Duvarci and Pare, 2014; Oliva et al., 2018
				*CeL*_*OFF*_−*CeM*	2.3	Haubensak et al., 2010; Mattera et al., 2020

The STDP synaptic modification rule is used in weights between the layers and the relative time between these layers' presynaptic and postsynaptic peaks. The values needed to parameterize the STDP equation are as follows: the time constant for weight adaptation is 10 τ, the time constant for long-term potentiation (LTP) is 10 τ_+_, and the time constant for long-term depression (LTD) is 0 τ_−_. The amplitude for LTP is 1.2 *A*_+_, and the amplitude for LTD is -0.4 *A*_−_.

The k-WTA function is used to determine hippocampal sparsity in the proposed model. The subregion *DG* receives 30% of the *EC*, *CA*3 receives 5% of the *DG*, and, finally, *CA*1 receives between 50% and 100%, in cases of stress elevation. *CA*3's recurring network is fully wired to help link parts of representation and retrieve patterns from memory. *CA*1 receives fully connected projection from *CA*3, and *ECV* has 50% sparsity.

Table 3 presents all input connections for each group of neurons used in the proposed model. Efforts were made to keep all values low while considering the proportional differences in neuron counts reported in the literature for various brain regions in rats (Boss et al., 1987; Gabbott et al., 1997; O'Reilly and Rudy, 2001; Maier and West, 2003; Chareyron et al., 2011). This approach maintains a realistic ratio of neurons across layers, ensuring both computational efficiency and biological plausibility.

**Table 3:** Synaptic inputs to each group of neurons.

**Region**	**Synaptic input**
*EC*_*II*_	*Context*·ω_*Context*−*E*_*C*__*II*__ + *RE*·ω_*RE*−*E*_*C*__*II*__ - *EC*_*II*_·ω_*E*_*C*__*II*_−*EC*_*II*__
*DG*	*EC*_*II*_·ω_*E*_*C*__*II*_−*DG*_ - *DG*·ω_*DG*−*DG*_
*CA*3	*EC*_*II*_·ω_*E*_*C*__*II*_−*CA*3_ + *DG*·ω_*DG*−*CA*3_ + *CA*3·ω_*CA*3−*CA*3_
*CA*1	*CA*3·ω_*CA*3−*CA*1_ + *RE*·ω_*RE*−*CA*1_ + *BA*_*F*_·ω_*B*_*A*__*F*_−*CA*1_ - *CA*_1_·ω_*C*_*A*__1_−*CA*_1__
*EC*_*V*_	*CA*_1_·ω_*C*_*A*__1_−*EC*_*V*__ + *EC*_*II*_·ω_*E*_*C*__*II*_−*EC*_*V*__ - *EC*_*V*_·ω_*E*_*C*__*V*_−*EC*_*V*__
*IL*	*PL*·ω_*PL*−*IL*_ + *BA*_*E*_·ω_*B*_*A*__*E*_−*IL*_ - *US*·ω_*US*−*IL*_
	+ *CA*_1_·ω_*C*_*A*__1_−*IL*_ + (1+β_*NE*_)·(*LC*·ω_*LC*−*IL*_)
*PL*	*RE*·ω_*RE*−*PL*_ - *CA*_1_·ω_*C*_*A*__1_−*PL*_ + *CA*_1_·ω_*C*_*A*__1_−*PL*_ + *BA*_*F*_·ω_*B*_*A*__*F*_−*PL*_
	+ (1+β_*NE*_)·(*LC*·ω_*LC*−*PL*_)
*RE*	*PL*·ω_*PL*−*RE*_ + *IL*·ω_*IL*−*RE*_ + *CA*_1_·ω_*C*_*A*__1_−*RE*_
*LA*	*CS*·ω_*CS*−*LA*_ + *CS*·ω_*CS*−*LA*_ - *LA*_*PV*_·ω_*L*_*A*__*PV*_−*LA*_
*LA*_*PV*_	*CS*·ω_*CS*−*L*_*A*__*PV*__ - *LA*_*CCK*_·ω_*L*_*A*__*CCK*_−*LA*_*PV*__
*LA*_*CCK*_	*US*·ω_*US*−*L*_*A*__*CCK*__
*BA*_*E*_	*IL*·ω_*IL*−*B*_*A*__*E*__ - *BA*_*CCK*_·ω_*B*_*A*__*CCK*_−*BA*_*E*__
*BA*_*CCK*_	*LA*·ω_*LA*−*B*_*A*__*CCK*__ + *BA*_*F*_·ω_*B*_*A*__*F*_−*BA*_*CCK*__
*BA*_*F*_	*LA*·ω_*LA*−*B*_*A*__*F*__ + *PL*·ω_*PL*−*B*_*A*__*F*__ + *CA*_1_·ω_*C*_*A*__1_−*BA*_*F*__
	- *BA*_*PV*_·ω_*B*_*A*__*PV*_−*BA*_*F*__ - *ITC*_*V*_·ω_*IT*_*C*__*V*_−*BA*_*F*__
*BA*_*PV*_	*BA*_*E*_·ω_*B*_*A*__*E*_−*BA*_*PV*__ + *IL*·ω_*IL*−*B*_*A*__*PV*__
*CeL*_*ON*_	*LA*·ω_*LA*−*Ce*_*L*__*ON*__
*CeL*_*OFF*_	- *CeL*_*ON*_·ω_*Ce*_*L*__*ON*_−*CeL*_*OFF*__ - *ITC*_*D*_·ω_*IT*_*C*__*D*_−*CeL*_*OFF*__
*ITC*_*D*_	*LA*·ω_*LA*−*IT*_*C*__*D*__ + *PL*·ω_*PL*−*IT*_*C*__*D*__
*ITC*_*V*_	*BA*_*E*_·ω_*B*_*A*__*E*3_−*ITC*_*V*__ + *IL*·ω_*IL*−*IT*_*C*__*V*__ - *ITC*_*D*_·ω_*IT*_*C*__*D*_−*ITC*_*V*__
*CeM*	*BA*_*F*_·ω_*B*_*A*__*F*_−*CeM*_ - *ITC*_*V*_·ω_*IT*_*C*__*V*_−*CeM*_ - *CeL*_*OFF*_·ω_*Ce*_*L*__*OFF*_−*CeM*_

## 3 Results

In this section, we expose the results achieved through the application of the proposed methodology, focusing on the evaluation of the neural network through models of contextual fear conditioning (CFC), immediate extinction deficit (IED), and stress-enhanced fear learning (SEFL).

### 3.1 Experimental consistency and data representation

To ensure the reliability of our experimental outcomes, we rigorously conducted mean and convergence analyses to determine the optimal number of repetitions for each condition, confirming that the observed patterns remained consistent and stable across trials. These analyses also helped establish the minimum number of repetitions required to produce consistent results (Note: Graphs illustrating these analyses are not included in this document). Using the well-known Contextual Fear Conditioning (CFC) paradigm, we observed that the graphical representations of the mean, boxplot, and confidence intervals of the means per number of repetitions began to converge from the fifteenth repetition. At this stage, the confidence intervals stabilized within an upper and lower limit of 5%. Consequently, a minimum of fifteen replicates was deemed sufficient to produce reliable and representative results for the experiments conducted in this study.

We visualize the data using boxplots, which effectively summarize the response distributions within each group. The boxplot format highlights central tendency and variability, allowing straightforward comparison across conditions. In these boxplots, the red “+” symbols indicate outliers-values that fall outside the expected range for each group. This visualization approach underscores the overall trends in the data and provides a solid basis for visually assessing group differences without the immediate need for statistical significance testing.

### 3.2 Contextual fear conditioning

**Figure 3:**
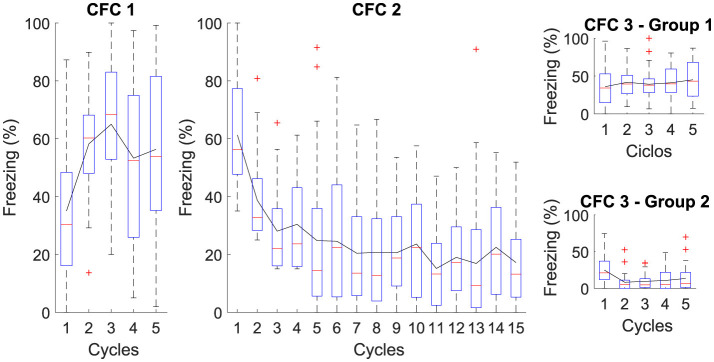
Average level of freezing (%) for Contextual Fear Conditioning: The Figure illustrates the sequential protocol of (CFC 1) fear acquisition, (CFC 2) fear extinction, (CFC 3) renewal, and repetition of fear extinction. In Phase CFC 1, the acquisition occurs for *AX*+. In Phase CFC 2, Context “B” for extinction is introduced, denoted as *BX*−. In Phase CFC 3, Group 1 presents renewal and Group 2 with repetition of extinction.

This experiment consists of three phases: Phase CFC 1, all groups were exposed to five cycles in *AX*+ for fear acquisition. Phase CFC 2 fifteen cycles of *BX*− for fear extinction. Phase CFC 3, Group 1, was reintroduced to *AX*− to assess fear renewal and Group 2 to *BX*− to assess repetition of extinction. This experiment analyses the process of fear extinction and examines the means of fear renewal after extinction. Table 4 details each step and Figure 3 outlines the maximum freezing level obtained for each phase.

**Table 4:** Contextual fear conditioning.

**Group**	**Phase CFC 1**	**Phase CFC 2**	**Phase CFC 3**
	**Day 1**	**Day 2**	**Day 3**
1	5*AX*+	15*BX*−	3*AX*−
2					3*BX*−

The symbols + and − represent the presence or absence of *US* (shock), respectively.

The figure illustrates the average freezing levels (%) across different phases of Contextual Fear Conditioning (CFC). In CFC 1, fear acquisition occurs with high freezing levels, indicating successful learning. In CFC 2, the introduction of a different context for extinction shows a gradual decrease in freezing, demonstrating effective fear extinction. CFC 3 presents two groups: Group 1 presents some fear renewal when the original context is reintroduced, while Group 2 exhibits further reduced freezing levels with repeated extinction. This reduction in freezing is attributed to continued extinction sessions, which result in the *CeM* receiving more inhibitory than excitatory signals, effectively suppressing the fear response.

The proposed neural architecture and computational modeling enable verifying that the extinction phase is essential to attenuate the association between context and fear, indicating that extinction reduces the existing fear response and makes it difficult to reactivate fear in the same context.

### 3.3 Fear response at different shock magnitudes during the acquisition phase

The following study investigates the mechanisms underlying the fear response at different shock magnitudes (SM) during the acquisition phase. Specifically, exploring the role of intensity in the conditioned stimulus-unconditioned stimulus (CS-US) pairing provides valuable information about how different threat levels modulate the fear response.

**Figure 4:**
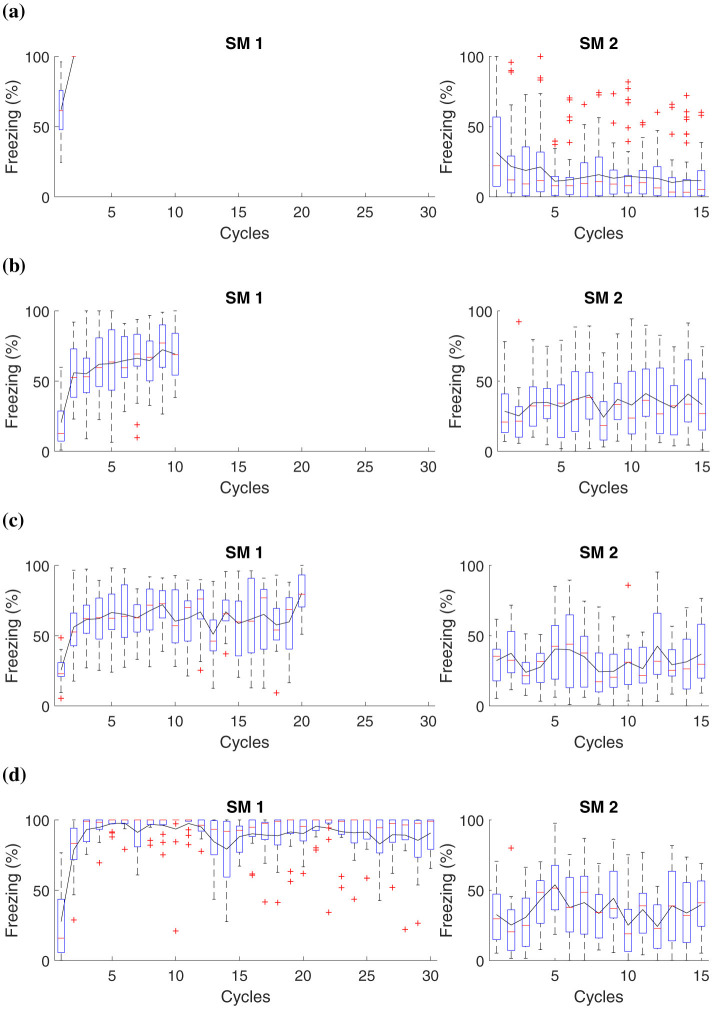
Average freezing level (%) for fear responses at different magnitudes during the acquisition phase. The Figure presents a simulation that captures fear responses at different magnitudes during the acquisition phase. In Phase SM-1, three distinct groups are subjected to different intensities of electric shock in *AX*+: **(A)** Group 1 receives a two shocks, **(B)** Group 2 receives ten shocks, **(C)** Group 3 receives twenty shocks, and **(D)** Group 4, thirty shocks. The simulation advances to Phase SM-2 with fifteen cycles in *BX*−.

For the simulation, each group, respectively, receives 1, 10, 20, and 30 shocks at the acquisition phase (Phase SM 1). The experiment features fifteen extinction cycles in Phase SM 2. Table 5 and Figure 4 present details of the experiments.

**Table 5:** Fear response at different shock magnitudes during the acquisition phase.

**Group**	**Phase SM 1**	**Phase SM 2**
	**Day 1**	**Day 2**
1	2*AX*+	15*BX*−
2	10*AX*+	15*BX*−
3	20*AX*+	15*BX*−
3	30*AX*+	15*BX*−

This experiment investigates and quantifies the intensity of the fear response at different magnitudes during the acquisition phase, allowing a more in-depth understanding of how subjects react variably to fear in the early stages of exposure or learning. The symbols + and − represent the presence or absence of *US* (shock), respectively.

This experiment demonstrates that when subjecting animals to shocks of low magnitude, as observed in groups that receive up to two shocks, the intensity may not be sufficient to establish a lasting aversive memory linked to the context or conditioned stimulus. Consequently, these animals demonstrate reduced fear retention.

The results suggest that after administering ten or more shocks, there is already a significant increase in fear retention, as evidenced by higher freezing responses. This marks a tipping point where the intensity and frequency of the unconditioned stimulus (the shocks) begin to consolidate a stronger aversive memory. Consequently, animals exhibit higher freezing rates during extinction, indicating substantial fear retention. Notably, after fifteen cycles, the stress response involving noradrenaline and corticosteroid levels, as described in the Neuromodulation section, becomes fully activated. These hormonal changes reinforce the established fear response, impacting the behaviors observed in Groups 2, 3, and 4 by enhancing alertness and stress regulation over time.

### 3.4 Stress-enhanced fear learning

**Figure 5:**
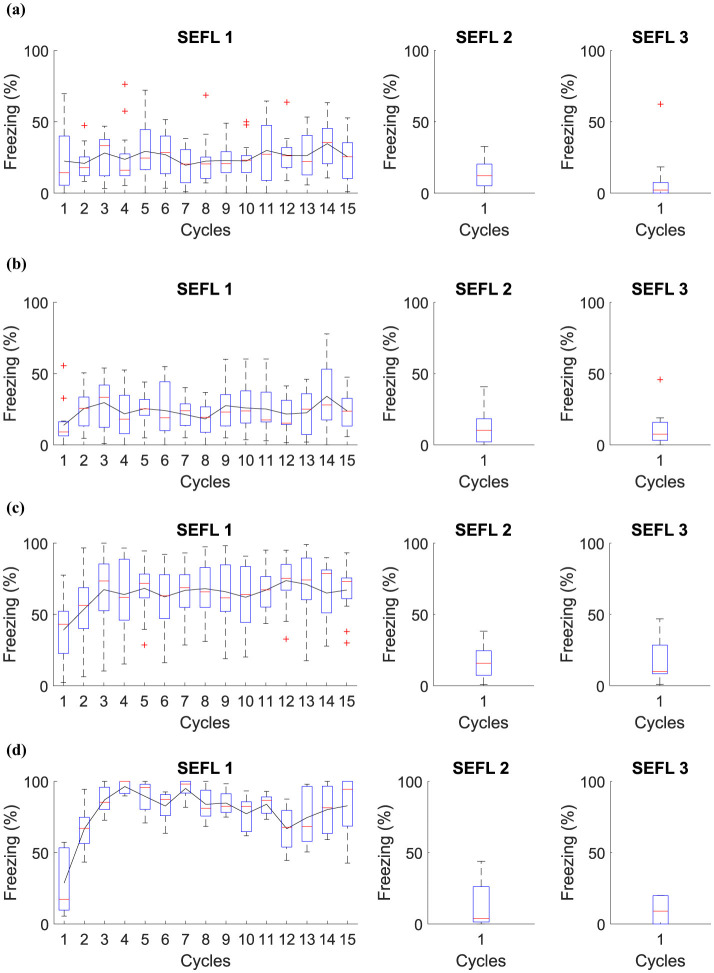
Average level of freezing (%) for fear responses obtained for SEFL. **(A)** Group 1 goes through Phase SEFL 1, fifteen cycles in *A*, in Phase SEFL 2, one cycle in *B*, and in Phase SEFL 3, one cycle in *B*. **(B)** Group 2 proceeds with Phase SEFL 1, fifteen cycles in *A*, Phase SEFL 2, one cycle in *B*+, and Phase SEFL 3, one cycle in *B*. **(C)** Group 3 experiences Phase SEFL 1, fifteen cycles in *A*+, Phase SEFL 2, one cycle in *B*, and Phase SEFL 3, one cycle in *B*. **(D)** Group 4 undergoes Phase SEFL 1, fifteen cycles at *A*+, Phase SEFL 2, one cycle at *B*+, and Phase SEFL 3, one cycle at *B*.

Based on the SEFL model, the following experiment investigates the induction of fear learning by stress. During Phase SEFL 1, Group 1, and Group 2 undergo 15 cycles in *A*, and Group 3 and Group 4 undergo fifteen cycles in *A*+. In Phase SEFL 2, Group 1, and Group 3 experience a cycle in *B*, and Group 2 and Group 4 are exposed to a cycle in *B*+. In Phase SEFL 3, all groups go through a cycle in *B*. The specific details of the experiment are elucidated in Table 6, while Figure 5 presents the collected data.

**Table 6:** Stress-Enduced Fear Learning.

**Group**	**Phase SEFL 1**	**Phase SEFL 2**	**Phase SEFL 3**
	**Day 1**	**Day 2**	**Day 3**
1	15*A*	1*B*	1*B*
2	15*A*	1*B*+	1*B*
3	15*A*+	1*B*	1*B*
4	15*A*+	1*B*+	1*B*

The symbols + represent the presence of *US* (shock), respectively.

The experiment reveals different results for each group studied, indicating variations in behavior and response to fear. Group 1 shows no significant changes in its behavior, suggesting a stable response to the experimental conditions. Group 2, on the other hand, exhibits higher freezing levels, a reaction that intensifies after being subjected to a shock in Context B.

Group 3 demonstrates a mild freezing reaction during the testing phase, which is notable considering that the shocks occurred in a context different from that used for testing. This analysis suggests a possible generalization of fear to different contexts.

Group 4 presents a significantly higher level of freezing. This group experienced a previous trauma in Context A and was likewise subjected to a shock in Context B the day before the test. This Group suggests that pre-existing fear, when combined with additional trauma, may result in a more pronounced fear response.

### 3.5 Shock stress must precede fear conditioning

This experiment analyzed whether previous and prolonged exposure to stress can increase fear responses. In Phase SS 1, all groups were inserted into Context *B*, with Group 3 and Group 4 receiving a shock. In Phase SS 2, Group 2 and Group 4 receive fifteen shocks in Context *A*, while Group 1 and Group 3 remain in context *A*. In Phase SS 3, all groups were submitted to Context *B* only once, and finally, in Phase SS 4, all groups are inserted into Context *A*.

**Figure 6:**
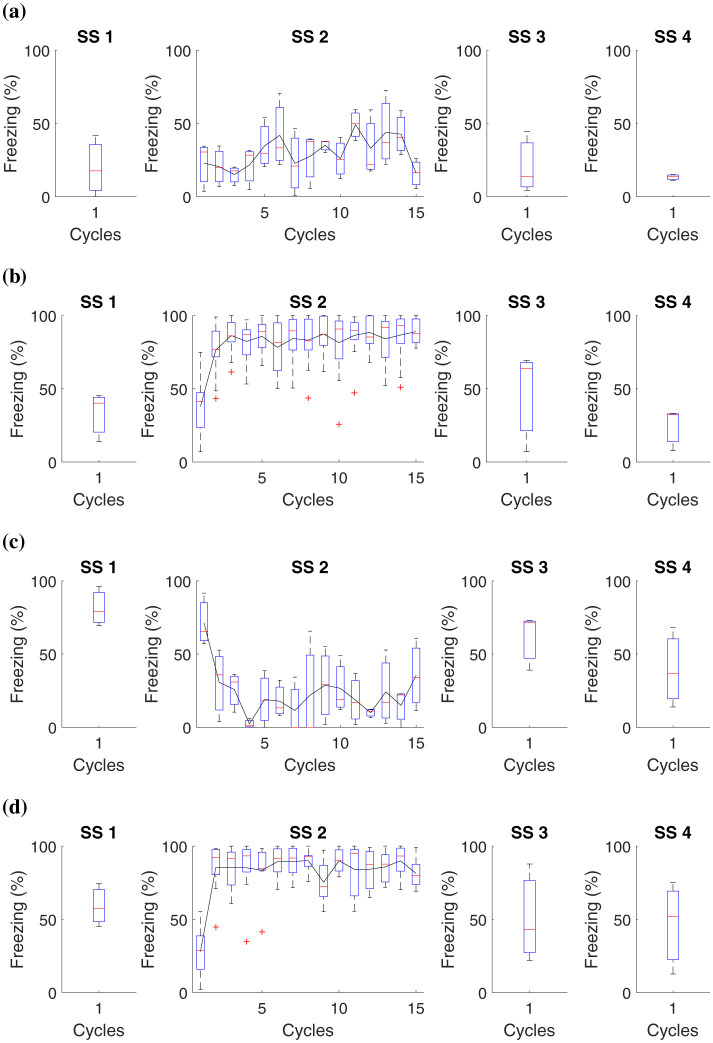
Average level of freezing (%) for fear responses obtained for “Shock stress (SS) must precede fear conditioning.” All groups undergo testing in Context *B* in Phase SS 3 and Context A in Phase SS 4. **(A)** Group 1 goes through Phase SS 1, one cycle in Context *B*, and Phase SS 2, 15 cycles in Context A. **(B)** Group 2 undergoes Phase SS 1, one cycle in Context *B*, and 15 cycles with shock in Context A. **(C)** Group 3 proceeds with Phase SS 1, one shock in Context *B*, and Phase SS 2, 15 cycles in Context A. **(D)** Group 4 experiences Phase SS 1, one shock in Context *B*, and Phase SS 2, 15 shocks in Context *A*.

The specific details of the experiment are elucidated in Table 7, while Figure 6 presents the collected data.

**Table 7:** Shock stress must precede fear conditioning.

**Group**	**Phase SS 1**	**Phase SS 2**	**Phase SS 3**	**Phase SS 4**
	**Day 1**	**Day 2**	**Day 3**	**Day 4**
1	1*B*	15*A*	1*B*	1*A*
2	1*B*	15*A*+	1*B*	1*A*
3	1*B*+	15*A*	1*B*	1*A*
4	1*B*+	15*A*+	1*B*	1*A*

The symbols + represent the presence of *US* (shock), respectively.

In this study, it was possible to observe that prolonged exposure to aversive stimuli, such as shocks, can increase an organism's tendency to develop more intense fear responses in future situations. Previous traumatic experiences amplify the learning process related to fear.

Data analysis revealed that, in animals subjected to a single shock in Context *B*, the fear reaction levels, measured through immobility behavior, were consistent regardless of having previously been exposed to multiple shocks in Context *A*. On the other hand, those who did not experience shocks in Context *A* presented minimal fear reactions in Context B. Experimental subjects who faced 15 shocks in Context A showed a high freezing reaction in this Context. However, this reaction was not altered by what occurred in Context *B*.

These results suggest that although previous traumatic experiences influence fear sensitivity, the specific fear response is more closely linked to the specific context where the aversive stimulus is experienced than to aversive experiences in different contexts.

### 3.6 Immediate Extinction Deficit

**Figure 7:**
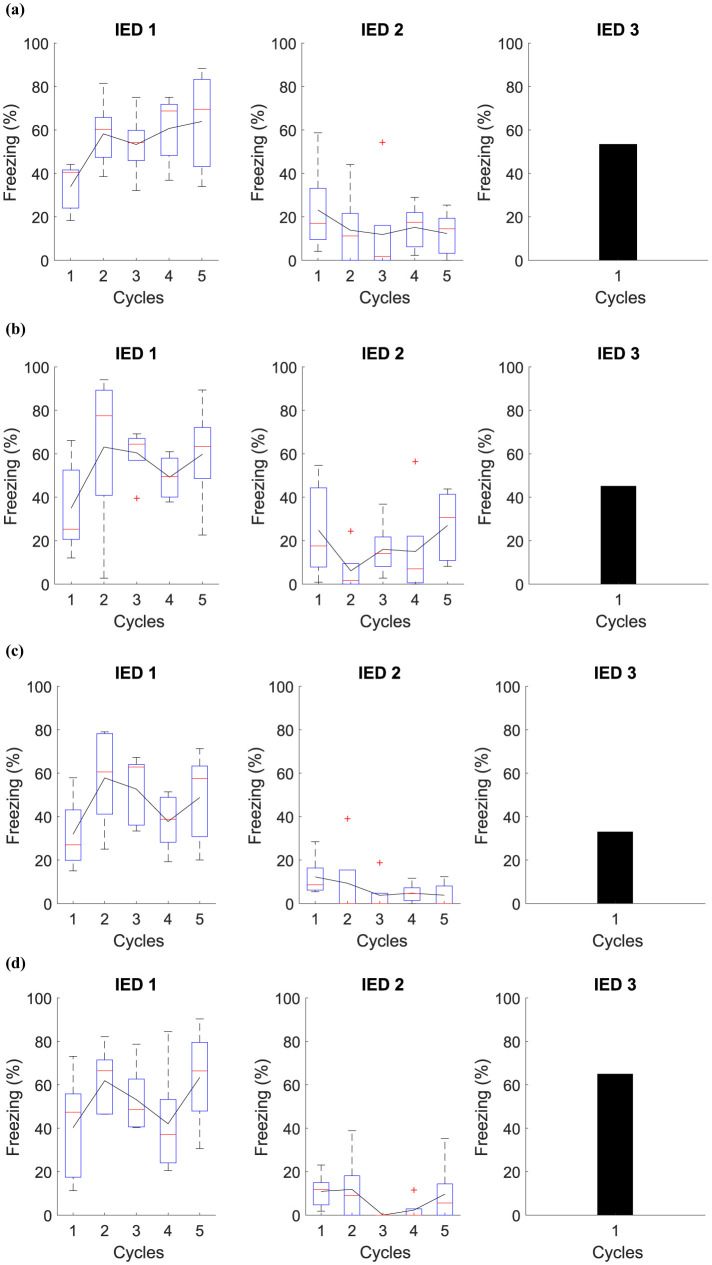
Average freezing level (%) for IED model. This experiment establishes the core parameters, centering on fear acquisition and extinction. It contrasts fear responses among **(A)** immediate, **(B)** delayed, **(C)** non-immediate, and **(D)** non-delayed extinction groups.

This experiment is based on the IED model and is used to analyze whether the timing of extinction influences the magnitude of fear in the fear retention phase. The experiment is divided into four groups: (1) immediate extinction, (2) delayed extinction, (3) immediate non-extinction, and (4) delayed non-extinction. Phase IED 1, all groups are exposed five times to Context *AX*+ to acquire fear. Phase IED 2, Group 1 undergoes five cycles in Context *BX*− 15 minutes after acquisition, while Group 2 receives the same five cycles in Context *BX*− but only 24 hours after acquisition. Group 3 goes through five cycles in Context *B* 15 minutes after acquisition, and Group 4 experiences the same five cycles in Context *B*, but 24 h after acquisition. After 48 hours of acquisition, all groups are exposed to three cycles in Context *CX*−. Table 8 presents the experiments related to the model, and Figure 7 presents the collected data.

**Table 8:** Immediate Extinction Deficit.

**Group**	**Phase IED 1**	**Phase IED 2**		**Phase IED 3**
	**Day 1**	**Day 1**	**Day 2**	**Day 3**
1	5*AX*+	5*BX*−	*Home*	3*CX*−
2	5*AX*+	*Home*	5*BX*−	3*CX*−
3	5*AX*+	5*B*	*Home*	3*CX*−
4	5*AX*+	*Home*	5*B*	3*CX*−

The symbols + and − represent the presence or absence of *US* (shock), respectively.

The IED model is critical for studying how the timing of fear extinction after its acquisition affects the longevity and magnitude of the fear response during subsequent retention tests. This insight is crucial for understanding potential therapeutic applications for conditions such as post-traumatic stress disorder, where fear memories can be persistently atypical or easily reactivated.

In our experiment, we observed that the average results between the groups were quite similar. However, we identified that Group 1 and Group 3 had a more significant freezing average than the other groups when placed in *CX*−. In this situation, the attempt at early extinction may interfere with the ability of the extinction memory to predominate over the fear memory. Furthermore, IED appears to be caused by a general fear response that does not depend on the specific environment where the animal learned the fear. Interestingly, Group 3 showed significantly lower freezing relative to the other groups.

By analyzing this and other experiments, it can be suggested that the stress caused by aversive stimuli during the acquisition of fear is the main cause of variations in how fear extinction occurs. Consequently, these variations can be attenuated by reducing the initially induced fear, either by performing fewer fear tests or by reducing the strength of the aversive stimuli. IED appears to be caused by a general fear response that does not depend on the specific environment where the animal learned the fear.

## 4 Discussion

Exposure therapy is essential in the treatment of psychiatric disorders. However, it faces the challenge of the fragile inhibitory memory of fear extinction, susceptible to relapses under stress or in the face of traumatic triggers. This fragility highlights the importance of reinforcing these inhibitory memories to reduce the risk of relapse and improve long-term therapeutic results. Consequently, it is vital to develop and refine experimental models that capture the complexity of the clinical scenario, enabling a deeper understanding of these disorders (Maren et al., 2013; Dunsmoor and Kroes, 2019; Sperandeo, 2013; Eichenbaum, 2000; Preston and Eichenbaum, 2013).

Consequently, our work developed a biologically and behaviorally plausible computational framework based on a rodent brain to analyze neural mechanisms related to fear and stress. The proposed architecture includes interactions between the amygdala, medial prefrontal cortex, nucleus reuniens, and hippocampus, incorporating data on stress hormones and how they directly affect these processes.

The model provides a structured framework to simulate and analyze distinct aspects of fear conditioning, IED, and SEFL. For fear conditioning, the model captures how the intensity and timing of aversive stimuli consolidate fear memories, influencing the retention and extinction of fear responses. Regarding IED, the model demonstrates how immediate extinction sessions post-fear acquisition can lead to generalized fear responses due to insufficient memory consolidation, highlighting the timing-dependent vulnerability of fear memory. For SEFL, the model simulates the sensitization process, where prior exposure to stress enhances fear learning in subsequent encounters, reflecting how repeated aversive experiences solidify fear memory networks and increase resistance to extinction. These mechanisms, simulated within the model, offer insights into the neural and hormonal pathways influencing each of these fear-related processes.

The selection of rats as the basis for the computational modeling in this study was grounded in the predominance of this species in fear conditioning protocols, such as Contextual Fear Conditioning, Stress-Enhanced Fear Learning (SEFL), and Immediate Extinction Deficit (IED). Rats possess complex brain structures and fear responses that closely resemble those observed in humans, making them ideal for detailed investigations of mechanisms related to fear and stress. Their use in these protocols ensures high biological fidelity, reinforcing the validity of the computational model for exploring experimental hypotheses and investigating behavioral dynamics with precision. Thus, choosing rats ensures the robustness and applicability of the model's results for studies simulating behavioral processes in fear contexts.

During the development of this study, extensive analyses and interpretations of computational simulations and biological modeling in existing literature were conducted. These analyses covered various aspects of fear reactions, stress, and their interrelationships, forming the basis of the proposed architecture. Previous models cited in this study, including those by Moustafa et al. (2009); John et al. (2013); Pendyam et al. (2013); Turnock and Becker (2008); Moustafa et al. (2013); Carrere and Alexandre (2015); Okon-Singer et al. (2015); Feng et al. (2016); Li (2017); Chang and Liang (2017); Mattera et al. (2020); Khalid et al. (2020); Kahana (2020), provided a crucial knowledge base for our experiments and performance of the model.

Our model aligns closely with established findings in the scientific literature on the neurobiology of fear and its extinction. It emphasizes the amygdala's pivotal role, particularly its lateral region (*LA*), as a central hub in processing conditioned and unconditioned stimuli, in agreement with studies by Akirav and Maroun (2007) and Carrere and Alexandre (2015). The *LA*'s extensive projections from sensory and associational cortices underscore its function as a primary site for encoding fear associations. Through this, the model reflects a current understanding that the *LA* is crucial for forming initial fear memories and plays a significant role in modulating the extinction process, as pathways within the amygdala are modified through repeated exposure to conditioned stimuli without reinforcement.

Furthermore, our model recognizes the central medial amygdala (*CeM*) as essential in orchestrating behavioral, autonomic, and endocrine responses associated with fear, which aligns with findings by Pape and Pare (2010) and Asede et al. (2015). The *CeM*'s output pathways influence brainstem structures that regulate physiological responses to fear, connecting emotional processing with somatic and autonomic functions. This integration within the model supports the *CeM*'s role in coordinating complex behavioral responses, a process that becomes especially relevant when considering heightened responses under stress.

The medial prefrontal cortex (*mPFC*) is another crucial component in our model, aligning with studies by Gilmartin et al. (2014) and Duvarci and Pare (2014). The *mPFC*'s modulatory influence on fear expression is modeled to reflect its dual role in both inhibiting and facilitating fear responses, depending on contextual cues and learning stages. The *mPFC*'s role in top-down regulation of the amygdala, particularly in extinguishing learned fear responses, is central to understanding adaptive responses and preventing generalized fear. This aspect of the model highlights how changes in *mPFC* activity can shift fear expression, a particularly relevant mechanism for therapeutic approaches targeting maladaptive fear.

Additionally, the model incorporates the hippocampus and entorhinal cortex, which are crucial for encoding and retrieving contextual fear memories. This inclusion aligns with research by Schapiro et al. (2017) and Maren et al. (2013), emphasizing the hippocampus's role in distinguishing between safe and threatening contexts. By accurately encoding environmental cues, the hippocampus enables differentiating responses based on context, which is essential for adaptive fear regulation. The entorhinal cortex facilitates this process by relaying spatial and contextual information to the hippocampus, further refining the response based on situational factors.

Our model also integrates the nucleus reuniens, which links cortical structures with the hippocampus and influences contextual fear learning and memory consolidation, as described in studies by Ramanathan et al. (2018). The nucleus reuniens uniquely integrates prefrontal inputs with hippocampal outputs, enhancing the model's capacity to simulate how contextual information modulates fear responses.

Finally, our model considers the activation of the sympathetic nervous system and the hypothalamic-pituitary-adrenal (HPA) axis in response to stressful events, in line with research by Drexler et al. (2019) and Grzelka et al. (2017). By incorporating hormonal responses, such as the release of corticosteroids and catecholamines, the model reflects the impact of stress on brain function, neuronal activity, and memory processing. Corticosteroids, in particular, are modeled to modulate synaptic plasticity, affecting how fear memories are consolidated and later retrieved. Catecholamines, such as norepinephrine, are included to capture their role in heightening arousal and alertness during fearful encounters. Together, these hormonal pathways interact with neural circuits to produce a multidimensional response to fear and stress, emphasizing the complexity of the extinction process and underscoring the intricate balance between physiological, neural, and hormonal influences in fear regulation.

### 4.1 Comparison with other models

In recent years, several computational models of different types have been proposed to study fear conditioning and extinction. Given the large output, this work focuses explicitly on models that closely align with the goals outlined by our research and that share similarities with the architecture we propose.

Firstly, among the computational works found and compatible with our work, none of them present tests for understanding stress (Moustafa et al., 2009; John et al., 2013; Turnock and Becker, 2008; Moustafa et al., 2013; Mattera et al., 2020; Khalid et al., 2020). However, each of them has critical objectives for fear literature. Like the works presented, our work can simulate conditioning, extinction, reacquisition, and fear renewal.

A distinction between our study and other works in the literature lies in the scope of the simulated brain regions and their objectives. Moustafa et al. (2009)'s study focuses on an update of a hippocampal model by Gluck and Myers (1993), adopting Hebbian learning and realistic stimulus representations. While Moustafa offers valuable insights into hippocampal learning and memory, our work encompasses a broader range of brain regions and interactions, providing a more holistic understanding of neural circuits. John et al. (2013) explores the hippocampus and prefrontal cortex interaction in regulating motivated behavior, focusing on contextual modulation and behavioral adaptation. While this model highlights the importance of memory and cognition, our study delves into the integration of additional regions and hormonal influence, which is crucial to understanding fear and stress.

Our computational model provides insights into the dynamics of aversive stimuli and their impact on fear acquisition and extinction. A critical factor is the intensity of the aversive stimulus in forming fear memory. We confirmed that higher intensity stimuli forge more robust and persistent fear memories, complicating the extinction process and aligning with previous findings (Rau et al., 2005). Additionally, the timing of post-acquisition extinction sessions is recognized as essential, as shown in studies by Kim et al. (2010) and Maren (2014). Immediate extinction following exposure may lead to greater fear generalization, suggesting that a consolidation period is necessary for fear memory to stabilize, enabling successful extinction effectively. Conversely, the absence of an extinction intervention reinforces the fear memory, strengthening the conditioned response.

The effects of stimulus intensity and extinction timing on fear memory have been extensively documented in the conditioning literature. High-intensity stimuli create more persistent fear memories that are harder to extinguish (Davis et al., 2000; Rescorla and Heth, 1975). Timing studies further demonstrate that immediate extinction can lead to generalized fear due to insufficient consolidation of fear memory (Quirk and Mueller, 2008; Maren and Holmes, 2016). Recent research also emphasizes the importance of precise timing, showing that interventions like vagus nerve stimulation must be carefully timed to enhance fear extinction in animal models (Souza et al., 2022). In addition, psychophysiological studies in humans confirm that intense stimuli produce more resilient fear memories, while appropriately timed extinction sessions improve fear reduction outcomes (Miller et al., 2023). These findings underscore the need to consider stimulus intensity and extinction timing in experimental designs investigating fear conditioning and extinction.

These results are significant in the context of cognitive disorders and illnesses, supporting the notion that stressful experiences exacerbate susceptibility to such conditions. This highlights the need for intervention strategies considering the intensity and timing of aversive stimuli. Through comparative analysis and results obtained in stress contexts, this study paves the way for developing new experiments that explore the interactions between fear and stress in the proposed neural architecture, enhancing significant advances in the field.

### 4.2 Limitations

The current model requires further refinement, including analyses of neuronal distribution across brain layers, precise determinations of synaptic weights (inhibitory and excitatory), and assessments of neuromodulatory changes. These steps are essential for a more detailed understanding of the neural mechanisms influencing emotional and cognitive disorders.

Another approach to enhancing the model would be to develop methods that capture individual variability in fear and stress responses. While our model primarily focuses on general mechanisms, it does not address how variations in biological responses, such as corticosteroid levels, may lead to different behavioral outcomes. Including such individual variability in future models could provide a more nuanced representation and enable predictions that account for individual biological and environmental differences.

## 5 Conclusion

The results of this study provide significant insights into the dynamics of fear acquisition, extinction, and reacquisition, as well as the differential impacts of stress on these processes. Through “Contextual Fear Conditioning” experiments, we demonstrate the intricate mechanisms by which fear memories are formed, extinguished, and potentially reactivated. Variations in fear responses due to different experimental conditions, such as the number of shock exposures and the timing of extinction protocols, underscore the complexity of emotional memory processes in the brain.

Our model allows us to carry out tests with the “Immediate Extinction Deficit” experiment, which confirms that the moment of fear extinction is crucial to determine the persistence and intensity of fear memories. This has profound implications for understanding the treatment of anxiety-related disorders, where the timing of therapeutic interventions can play a critical role in their effectiveness.

Furthermore, testing the model in the “Stress-Enhanced Fear Learning” experiment confirms the significant role of pre-existing stress in enhancing fear memory formation. This observation is particularly relevant in the context of stress disorders, suggesting that prior exposure to stress may exacerbate fear responses in subsequent fear-inducing situations.

The study also highlights the need for personalized approaches in therapeutic interventions for conditions related to fear and stress. Understanding individual variability in response to fear and stress, as indicated by our experiments' varying levels of freezing behavior, is crucial for developing more targeted and effective treatments.

These results contribute to a deeper understanding of the neurobiological underpinnings of fear and stress responses. They pave the way for future research, particularly in exploring the potential of personalized therapy in treating anxiety disorders and PTSD and in understanding the broader implications of stress on cognitive and emotional health. Integrating these findings into clinical practice could significantly increase the effectiveness of treatments for a wide range of psychological conditions related to fear and stress.

## Data Availability

The original contributions presented in the study are included in the article/supplementary material, further inquiries can be directed to the corresponding author.
